# Surgical excision margins for primary cutaneous melanoma

**DOI:** 10.1590/S1516-31802011000100013

**Published:** 2011-01-06

## Abstract

**ABSTRACT:**

**BACKGROUND:**

Cutaneous melanoma accounts for 75% of skin cancer deaths. Standard treatment is surgical excision with a safety margin some distance from the borders of the primary tumour. The purpose of the safety margin is to remove both the complete primary tumour and any melanoma cells that might have spread into the surrounding skin. Excision margins are important because there could be trade-off between a better cosmetic result but poorer long-term survival if margins become too narrow. The optimal width of excision margins remains unclear. This uncertainty warrants systematic review.

**OBJECTIVES:**

To assess the effects of different excision margins for primary cutaneous melanoma.

**SEARCH STRATEGY:**

In August 2009 we searched for relevant randomised trials in the Cochrane Skin Group Specialized Register; the Cochrane Central Register of Controlled Trials (CENTRAL) in The Cochrane Library (Issue 3, 2009), Medline, Embase, Lilacs, and other databases including Ongoing Trials Registers.

**SELECTION CRITERIA:**

We considered all randomized controlled trials (RCTs) of surgical excision of melanoma comparing different width excision margins.

**DATA COLLECTION AND ANALYSIS:**

We assessed trial quality, and extracted and analyzed data on survival and recurrence. We collected adverse effects information from included trials.

**MAIN RESULTS:**

We identified five trials. There were 1633 participants in the narrow excision margin group and 1664 in the wide excision margin group. Narrow margin definition ranged from 1 to 2 cm; wide margins ranged from 3 to 5 cm. Median follow-up ranged from 5 to 16 years.

**AUTHORS' CONCLUSIONS:**

This systematic review summarises the evidence regarding width of excision margins for primary cutaneous melanoma. None of the five published trials, nor our meta-analysis, showed a statistically significant difference in overall survival between narrow or wide excision. The summary estimate for overall survival favoured wide excision by a small degree [Hazard Ratio 1.04; 95% confidence interval 0.95 to 1.15; P = 0.40], but the result was not significantly different. This result is compatible with both a 5% relative reduction in overall mortality favouring narrower excision and a 15% relative reduction in overall mortality favouring wider excision. Therefore, a small (but potentially important) difference in overall survival between wide and narrow excision margins cannot be confidently ruled out. The summary estimate for recurrence free survival favoured wide excision [Hazard Ratio 1.13; P = 0.06; 95% confidence interval 0.99 to 1.28] but again the result did not reach statistical significance (P < 0.05 level). Current randomized trial evidence is insufficient to address optimal excision margins for primary cutaneous melanoma.

**COMMENTS:**

This paper makes it clear that there is no proven statistically significant difference in overall survival or recurrence-free survival, between wide margins (3-5 cm) and narrow margins (1-2 cm) for treating cutaneous melanoma. At this moment, minimum margins of 1.0 cm and maximum margins of 2.0 cm are considered appropriate for specific anatomical situations, without compromising the cure or quality of life of these patients. It has to be borne in mind that even though most studies in the literature only evaluated the prognostic factor of “thickness”, it is now well known that other prognostic factors such as sentinel node appearance, anatomical location and cytogenetic parameters need to be considered in proposals for future randomized studies aimed towards defining ideal margins.
